# Feature selective temporal prediction of Alzheimer's disease progression using hippocampus surface morphometry

**DOI:** 10.1002/brb3.733

**Published:** 2017-06-09

**Authors:** Sinchai Tsao, Niharika Gajawelli, Jiayu Zhou, Jie Shi, Jieping Ye, Yalin Wang, Natasha Leporé

**Affiliations:** ^1^ CIBORG Children's Hospital Los Angeles and University of Southern California Los Angeles CA USA; ^2^ Department of Computer Science and Engineering Michigan State University East Lansing MI USA; ^3^ Department of Computational Medicine and Bioinformatics & Department of Electrical Engineering and Computer Science University of Michigan Ann Arbor MI USA; ^4^ School of Computing, Informatics and Decision Systems Engineering Arizona State University Phoenix AZ USA

**Keywords:** Alzheimer's Disease, dementia, hippocampus, machine learning, multi‐task learning, tensor‐based morphometry

## Abstract

**Introduction:**

Prediction of Alzheimer's disease (AD) progression based on baseline measures allows us to understand disease progression and has implications in decisions concerning treatment strategy. To this end, we combine a predictive multi‐task machine learning method (cFSGL) with a novel MR‐based multivariate morphometric surface map of the hippocampus (mTBM) to predict future cognitive scores of patients.

**Methods:**

Previous work has shown that a multi‐task learning framework that performs prediction of all future time points simultaneously (cFSGL) can be used to encode both sparsity as well as temporal smoothness. The authors showed that this method is able to predict cognitive outcomes of ADNI subjects using FreeSurfer‐based baseline MRI features, MMSE score demographic information and ApoE status. Whilst volumetric information may hold generalized information on brain status, we hypothesized that hippocampus specific information may be more useful in predictive modeling of AD. To this end, we applied a multivariate tensor‐based parametric surface analysis method (mTBM) to extract features from the hippocampal surfaces.

**Results:**

We combined mTBM features with traditional surface features such as middle axis distance, the Jacobian determinant as well as 2 of the Jacobian principal eigenvalues to yield 7 normalized hippocampal surface maps of 300 points each. By combining these 7 × 300 = 2100 features together with the previous ~350 features, we illustrate how this type of sparsifying method can be applied to an entire surface map of the hippocampus that yields a feature space that is 2 orders of magnitude larger than what was previously attempted.

**Conclusions:**

By combining the power of the cFSGL multi‐task machine learning framework with the addition of AD sensitive mTBM feature maps of the hippocampus surface, we are able to improve the predictive performance of ADAS cognitive scores 6, 12, 24, 36 and 48 months from baseline.

## 1 INTRODUCTION

Recent work in psychological testing (Caselli et al., 2013), genetic studies (Elias‐Sonnenschein et al., 2013), magnetic resonance (MR) imaging (Teipel et al., 2013), positron emission tomography (PET) imaging (Becker et al., 2013), cerebral spinal fluid (CSF) measurements (Blennow & Zetterberg, 2013), cardiovascular status (Hajjar, Brown, Mack, & Chui, 2013) and others have yielded tremendous amounts of diagnostic data for diagnosing and staging dementias, especially Alzheimer's disease (AD). Moreover, many of these studies now also include longitudinal information (Caselli et al., 2013; Mueller et al., 2005). This has led to a problem often referred to as the ‘curse of dimensionality’, where the size (number of dimensions) of the dataset makes it difficult to perform numerical analyses on the data. This in turn makes it increasingly difficult to draw consistent conclusions from the dataset. Traditional approaches to dimension reduction eliminates variables / dimensions based on clinical assumptions and allows us to test specific hypothesis about the disease model. However, it does not lend itself to discovering new correlations or allow for all inclusive models that are consistent across all dimensions. These problems become even more important when trying to improve predictions using machine learning techniques. This is mainly because at a point the predictive power of the model ceases to increase by just adding more information or dimensions. The question is then about how to select the “correct” features to maximize predictive power. Zhou, Liu, Narayan, Ye, and Ye (2013) outlines a method that simultaneously enforces low dimensionality through sparsity of weights and temporal smoothness of the predicted behavioral scores at 6, 12, 24, 36 and 48 months. This paper leverages this method, built specifically for progressive disease models, such as AD, together with multivariate tensor‐based morphometric (mTBM) features (Wang, Yuan, et al., 2010) of the hippocampus to predict AD progression up to 48 months from the baseline MRI measurement. The goal is to evaluate the predictive power of mTBM against those of cortical thickness and other FreeSurfer‐based features, demographic information (sex and age) as well as genetic information (ApoE‐ε4 Copies).

Alzheimer's Disease is characterized by non‐focal deterioration of brain tissue and many attempts have been made at imaging this phenomenon. This includes the use multiple modalities including CT, PET and MRI. PET has been a powerful technique for imaging AD, especially with the development of the Pittsburgh Compound B (PiB) tracer that enhances beta‐amyloid plaques (Klunk et al., 2004). However, MRI is more commonly used because of the lack of ionizing radiation and good white matter / grey matter tissue contrast. MR also allows for multiple image contrasts to be generated in a single session. T1‐weighted high resolution structural images have revealed widespread atrophy of the both white matter and gray matter tissues. In particular, the deep gray matter structures – particularly, the hippocampus ‐ correlate strongly with AD progression (Barber, Ballard, McKeith, Gholkar, & O’ Brien, 2000; Bozzali, Franceschi, Falini, & Pontesilli, 2001; Jack, Shiung, Gunter, & O’ Brien, 2004; Jack et al., 1999; Killiany, Hyman, Gomez‐Isla, & Moss, 2002; Petersen, Jack, Xu, Waring, & O’ Brien, 2000; deToledo‐Morrell et al., 2004; Xu, Jack, O’ Brien, Kokmen, & Smith, 2000). Similarly, diffusion weighted imaging has revealed disruption of a number of crucial white matter tracts associated with the limbic system (Bozzali, Falini, & Franceschi, 2002; Bozzali et al., 2001; Choi, Lim, & Monteiro, 2005; Chua, Wen, & Slavin, 2008; Clerx, Visser, Verhey, & Aalten, 2012; Concha, Gross, & Beaulieu, 2005; Douaud et al., 2011; Frisoni, Fox, Jack, & Scheltens, 2010; Jack, Bernstein, & Fox, 2008; Jahng et al., 2011; Lo, Wang, Chou, & Wang, 2010; Nakata et al., 2009; Rose, Chen, Chalk, & Zelaya, 2000; Sexton, Kalu, Filippini, & Mackay, 2011; Takahashi, Yonezawa, Takahashi, & Kudo, 2002; Yoshiura, Mihara, Ogomori, & Tanaka, 2002; Zhang, Schuff, Ching, & Tosun, 2011; Zhang, Schuff, Du, Rosen, & Kramer, 2009; Zhang, Schuff, Jahng, Bayne, & Mori, 2007). Functional connectivity MRI has also shown decreases in the default mode as well as other brain networks. Clinically, the current AD diagnosis criteria include the use of (1) MRI, (2) PET as well as (3) beta‐amyloid load within the cerebral spinal fluid (McKhann, Knopman, & Chertkow, 2011; Ray, Britschgi, Herbert, & Takeda‐Uchimura, 2007). To measure severity of dementia, tests such as MMSE and CDR are often used (Tan:2011vt, OBryant:2008bk, Morris:1997vu).

As MR imaging has become more ubiquitous as a research and clinical tool, there has been an effort in developing image‐based features that are increasingly sensitive to AD progression as well as the conversion from Mildly Cognitively Impaired (MCI) to AD. Early attempts used volumetric measurements of tissue types (WM or GM) and then the volume of specific structures such as the hippocampus (De Jong, Van der Hiele, Veer, & Houwing, 2008; Fox, Warrington, & Freeborough, 1996; Frisoni et al., 2010; Jack et al., 2008; Laakso, Partanen, Riekkinen, & Lehtovirta, 1996; Ridha, Barnes, Bartlett, & Godbolt, 2006; Scahill, Schott, & Stevens, 2002; Schuff, Woerner, Boreta, Kornfield, & Shaw, 2009). Attempts were also made at quantifying the degree of deformation associated with the atrophying demented brain using tensor‐based morphometric (TBM) techniques (Baron, Chetelat, Desgranges, & Perchey, 2001; Grossman, McMillan, Moore, Ding, & Glosser, 2004; Hirata, Matsuda, Nemoto, Ohnishi, & Hirao, 2005; Hua, Leow, Parikshak, Lee, & Chiang, 2008; Hua et al., 2009; Hua et al., 2008; Karas, Burton, Rombouts, & Van Schijndel, 2003; Lerch, Pruessner, Zijdenbos, & Hampel, 2005; Oishi et al., 2009; Salat, Buckner, Snyder, & Greve, 2004; Teipel, Born, Ewers, Bokde, & Reiser, 2007; Thompson, Hayashi, Sowell, & Gogtay, 2004). In addition to volumetric deformations, (Shi, Thompson, et al. 2013) applied multivariate TBM (mTBM) to the hippocampus surface and showed marked improvement in sensitivity of detecting AD progression.

At the same time, the machine learning community recognized the utility in predicting disease progression as a means of characterizing AD disease progression. It allows for an inclusive look at how the different diagnostic indicators account for observed changes. However, researchers were faced with finding selecting meaningful features to be used as well as how to incorporate data with multiple time points (Davatzikos, Fan, Wu, Shen, & Resnick, 2008; Klöppel, Stonnington, Chu, & Draganski, 2008; Lao, Shen, Xue, Karacali, & Resnick, 2004; Li, Shi, Pu, Li, & Jiang, 2007; Magnin, Mesrob, & Kinkingnéhun, 2009; Morra, Tu, Apostolova, & Green, 2008; Shankle, Mani, Pazzani, & Smyth, 1997; Stonnington, Chu, Klöppel, & Jack, 2010; Sun, van Erp, Thompson, & Bearden, 2009; Trambaiolli, Lorena, & Fraga, 2011; Vemuri, Gunter, Senjem, & Whitwell, 2008; Ye, Wu, Li, & Chen, 2011; Zhang & Shen, 2012; Zhang, Wang, Zhou, Yuan, & Shen, 2011). (Zhou et al., 2013) tackled this problem by using a convex fused sparse group lasso (cFSGL) framework that incorporated temporal smoothness to predict disease progression as measured by MMSE and CDR. Generic volumetric and cortical thickness generated by freesurfer was used as imaging features in addition to a host of other clinical descriptors.

However, combining cFSGL with a more AD specific / sensitive features such as surface deformations fields of the hippocampus might improve the predictive power of the algorithm significantly. To this end, we augmented the generic FreeSurfer‐based image features with novel mTBM features of the hippocampus and other surface deformation field based features (see Table 1 for features), which significantly increased the predictive power of the cFSGL technique.

**Table 1:** List of original features from (Zhou et al., 2013) and new surface features (downsized by 10) computed from the hippocampus used to predict outcomes at 6, 12, 24, 36 and 48 months

	No of features	
Original features		
Sex	1	309
Age	1	
ApoE	1	
Baseline MMSE	1	
MRI features: (average cortical thickness, standard deviation in cortical thickness, the volumes of cortical parcellations (based on regions of interest automatically segmented in the cortex), the volumes of specific white matter parcellations, and the total surface area of the cortex.	305	
Hippocampal surface features		
Mid Axis Distance map	300	
mTBM feature maps (3 tensor values × 300 points)	900	2100
Jacobian magnitude map	300	
Jacobian principal eigen values (2 × 300 points)	600	

## 2 METHODS

### 2.1 ADNI data

Data used in the preparation of this article were obtained from the Alzheimer's Disease Neuroimaging Initiative (ADNI) database (adni.loni.usc.edu). ADNI was launched in 2003 by the National Institute on Aging (NIA), the National Institute of Biomedical Imaging and Bioengineering (NIBIB), the Food and Drug Administration (FDA), private pharmaceutical companies and non‐profit organizations, as a $60 million, 5‐year public‐ private partnership. The primary goal of ADNI has been to test whether serial magnetic resonance imaging (MRI), positron emission tomography (PET), other biological markers, and clinical and neuropsychological assessment can be combined to measure the progression of mild cognitive impairment (MCI) and early Alzheimer's disease (AD). Determination of sensitive and specific markers of very early AD progression is intended to aid researchers and clinicians to develop new treatments and monitor their effectiveness, as well as lessen the time and cost of clinical trials.

The Principal Investigator of this initiative is Michael W. Weiner, MD, VA Medical Center and University of California – San Francisco. ADNI is the result of efforts of many co‐ investigators from a broad range of academic institutions and private corporations, and subjects have been recruited from over 50 sites across the U.S. and Canada. The initial goal of ADNI was to recruit 800 subjects but ADNI has been followed by ADNI‐GO and ADNI‐2. ADNI‐GO or “Grand Challenges” and ADNI‐2 supplements ADNI by trying to identify patients in the pre‐dementia or early mildly cognitively impaired (eMCI) phase. To date these three protocols have recruited over 1500 adults, ages 55 to 90, to participate in the research, consisting of cognitively normal older individuals, people with early or late MCI, and people with early AD. The follow up duration of each group is specified in the protocols for ADNI‐1, ADNI‐2 and ADNI‐GO. Subjects originally recruited for ADNI‐1 and ADNI‐GO had the option to be followed in ADNI‐2. For up‐to‐date information, see www.adni-info.org.

For our experiment we used 616 subjects from ADNI‐1 where we had 606 subjects that have behavioral scores for M06, 606 for M12, 533 for M24, 364 for M36 and 97 for M48. Zhou et al. (2013) methods allows us to train prediction using data that have missing time points, so subjects that has missing time points can be used. 90% of the data was used for training and 10% used for testing. The reported results are for 20 different selection splits of training and testing. More information about the demographics and patient selection is available in (Zhou et al., 2013).

### 2.2 Freesurfer MRI features

The MRI image analysis software Freesurfer (Fischl, 2012) was used to extract 305 MRI features based on cortical reconstruction and volumetric segmentations. The features can be group into 5 categories: average cortical thickness, standard deviation in cortical thickness, the volumes of cortical parcellations (based on regions of interest automatically segmented in the cortex), the volumes of specific white matter parcellations, and the total surface area of the cortex. This process was performed by the ADNI team at UCSF under the ADNI harmonized MRI processing protocols as outlined on their website (http://adni.loni.usc.edu/methods/mri-analysis/). See Table 1 for a more complete feature list and breakdown.

### 2.3 Hippocampus surface computation

The details of the entire methodology of extracting mTBM features from surface registered hippocampal maps is outlined in Shi, Thompson, et al. (2013), we have outlined the key steps of the method in this paper. FSL's (Jenkinson, Beckmann, & Behrens, 2012) automated segmentation program FIRST was used to segment the MRI volumes to extract binary volumes for the hippocampus. The surfaces were then computed by running a topology‐preserving level set method (Han, Xu, & Prince, 2003) to ensure the segmentation was topological correct before tessellation via a marching cubes algorithm (Lorensen & Cline, 1987).

### 2.4 Conformal representation and surface registration of the hippocampus

**Figure 1:**
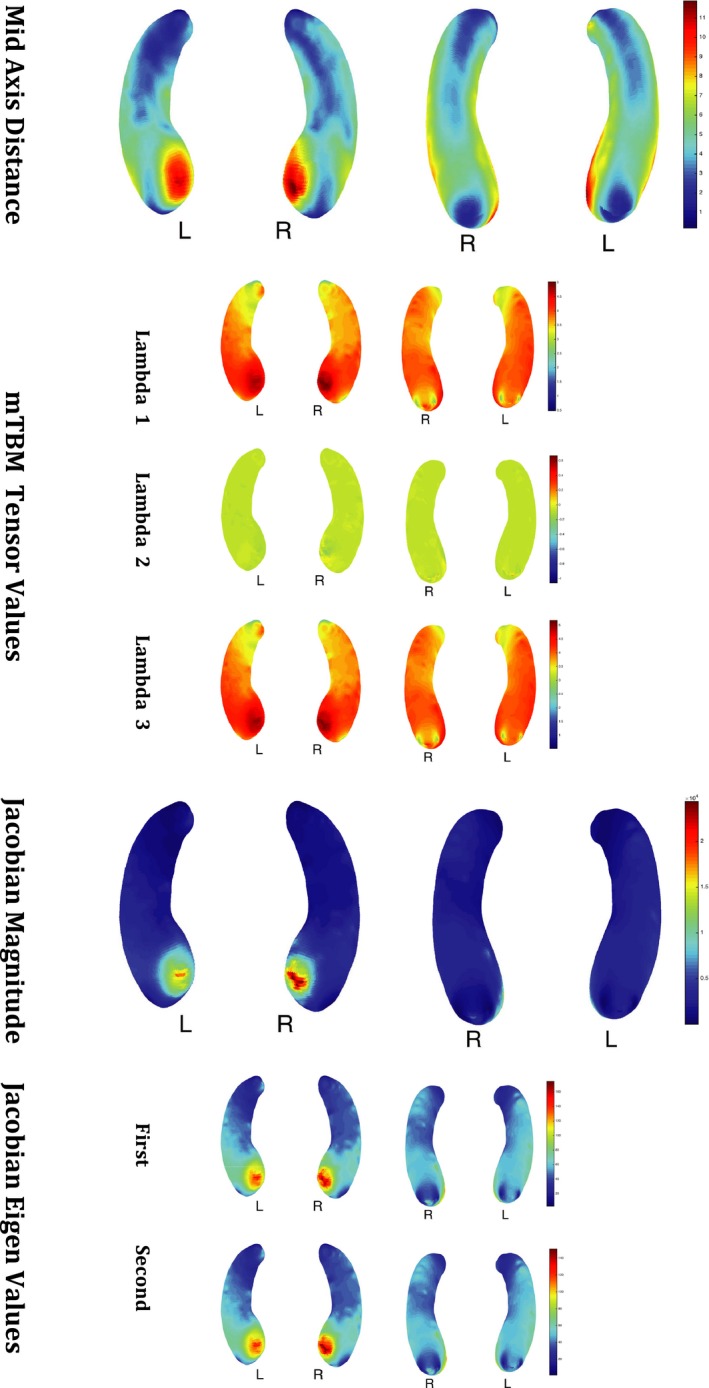
Example of Feature Maps of the Hippocampus for 1 subject

In order for discretized imaging data to be used in group analysis and prediction tasks, they must be transformed into a common space that allows for one‐to‐one correspondence across subjects. Examples of the mean hippocampal common space can be seen in Figure 1. In our case, we would like to use measurements on a discretized surface represented my vertices in $R^{3}$ and edges between the vertices. In this case, we first conformally mapped the hippocampal surface onto a rectangular plannar surface using holomorphic 1‐forms. The surface conformal representation is then computed using the local conformal factor as well as mean curvature. The dynamic range of the conformal representation is then linearly scaled to form the feature image of the surface. The feature image aligned with a template image via fluid registration in a curvilinear coordinate system that compensates for distortions due to the conformal parameterization (Shi, Thompson, et al. 2013). There are numerous advantages of using conformal representation with fluid registration to align the hippocampal surfaces: (1) the entire transform is diffeomorphic and therefore has diffeomorphic shape correspondences that are smooth and one‐to‐one. (2) The transform is inverse consistent and therefore more robust than unidirectional transformations (Leow et al., 2005). (3) Because conformal parametrization induces a simple Riemannian metric, the Navier‐Stokes equation in the fluid registration can be easily adjusted for area distortion (Wang, Chiang, & Thompson, 2005a,b).

### 2.5 Multivariate tensor‐based morphometry (mTBM)

After automatically segmenting hippocampus with FSL (Jenkinson et al., 2012) from brain MR images, we build parametric meshes to model hippocampal shapes. High‐order correspondences between hippocampal surfaces were enforced across subjects with a novel inverse consistent surface fluid registration method. Multivariate statistics consisting of multivariate tensor‐based morphometry (mTBM) and radial distance were computed for surface deformation analysis (Shi, Thompson, et al. 2013; Wang, Yuan, et al., 2010). Multivariate tensor‐based morphometric (mTBM) analysis has been used as a sensitive method of comparing deformation fields of different subjects with the aim of discovering group‐wise differences (Lepore et al., 2008; Wang, Zhang, et al., 2010). mTBM generates Riemannian manifolds from the full deformation fields that map each subject to the template space and statistics are computed on these manifolds. Specifically, compared to univariate TBM which uses the Jacobian of the transformation that mainly describes the volumetric changes, mTBM uses the full deformation information by applying a manifold version of Hotelling's test to Riemannian manifolds in log‐euclidean space. The idea is to be able to describe higher order transformations with a single metric instead of using derived metrics from the Jacobian (see Figure 1 for examples of mTBM features).

Shi et al. 2013 showed that a surface derived from a reasonable segmentation using FSL is sensitive enough to detect group‐wise differences in the mTBM features. Moreover, mTBM is also more statistically sensitive with better power as shown by false discovery rates (Lepore et al., 2008). In this work, we've added these sensitive features to the existing MR‐based surface area and volumetric features to boost AD prediction accuracy.

### 2.6 Convex fused sparse group lasso

Zhou et al. (2013) proposed a powerful multi‐tasked learning technique that incorporates sparsity as well as temporal smoothing for modeling a progressive disease model. In their formulation, each tasked can be though of a single forward predictor from baseline measurement to a measurement at a certain future time point. In their case, they used the ADNI dataset and predicted ADAS cognitive scores 6 months after baseline (M06), 12 months after baseline (M12), 24 months after baseline (M24), 36 months after baseline (M36) and 48 months after baseline (M48). In our study we aim to use the same ADNI dataset but also incorporate 7 hippocampus surface feature maps of 300 points (2100 features total) and compare it to the predictive performance of using only simple regional volumes and surface areas used (305 features total) in their study.

The cFSGL method that we use can be considered a multi‐task regression problem with ***t*** time points and from ***n*** subjects each with ***d*** features, where $\left\{x_{1},\;x_{2},\ldots ,\;x_{n}\right\}$ represents each of the ***d*** input features for each subject at baseline (i.e. $x_{i}\in R^{d}$ ). Similarly, $\left\{y_{1},\;y_{2},\ldots ,\;y_{n}\right\}$ represents the target cognitive scores for each subject at **T** time points (i.e. $y_{i}\in R^{t}$). For a single subject (***n***), each task can be seen as a projection of MR / demographic / genetic baseline measurements at *t* = 0 represented as ***x***_*n*_ to a future cognitive score measurement at time *t* = *t*_1_ (e.g. at 48 months) given by the appropriate row in vector ***y***_*n*_. We can extend this formulation to a multi‐task one by performing projections of all time points simultaneously. In other words, each set of baseline measurements for a single subject at *t* = 0 given by ***x***_1_ ($R^{d}$ with ***d*** features) is projected to a vector ($R^{t}$ with ***T*** time points) given by ***y***_1_. The entire population‐based mapping can be summarized as a linear operation using matrices ***X*** and ***Y***. ***X*** and ***Y*** are formed by arranging the input and output patient feature space row‐wise, each row being ***x***_*n*_ or ***y***_*n*_, (i.e. $X={\left[x_{1},\;x_{2},\ldots ,\;x_{n}\right]}^{T},Y={\left[y_{1},\;y_{2},\ldots ,\;y_{n}\right]}^{T}$) and yield a $X\in R^{n\times d}$ matrix and a $Y\in R^{n\times t}$ matrix. Since this is a linear model, a set of weights $W={\left[w^{1},\;w^{2},\ldots ,\;w^{t}\right]}^{T}\in R^{d\times t}$ is trained to map ***x***_*n*_ to ***y***_*n*_ or ***X*** to ***Y***.

$$\left[\begin{array}{ccc} x_{1}^{1} & \cdots & x_{1}^{d}\\ \vdots & \ddots & \vdots \\ x_{n}^{1} & \cdots & x_{n}^{d} \end{array}\right]\times \left[\begin{array}{ccc} w_{1}^{1} & \cdots & w_{1}^{t}\\ \vdots & \ddots & \vdots \\ w_{d}^{1} & \cdots & w_{d}^{t} \end{array}\right]=\left[\begin{array}{ccc} y_{1}^{1} & \cdots & y_{1}^{t}\\ \vdots & \ddots & \vdots \\ y_{n}^{1} & \cdots & y_{n}^{t} \end{array}\right]$$

To achieve a set of weights that encodes both sparsity and temporal smoothness, the following cost function is minimized during training:

$$\min_{W}||XW-{Y||}_{F}^{2}+\lambda_{1}\left|\right|{W||}_{1}+\lambda_{2}\left|\right|{RW}^{T}{\left|\right|}_{1}+\lambda_{3}\left|\right|{W||}_{2,1}$$

where ||***W***_1_|| is the L1‐norm or lasso penalty that encodes for sparsity, $\left|\right|W_{2,1}\left|\right|=\sum_{i=1}^{d}\sqrt{\sum_{j=1}^{t}W_{ij}^{2}}$ is the group Lasso penalty that encodes for temporal grouping of features.

$\left|\right|{RW}^{T}{\left|\right|}_{1}$ is the fused lasso penalty as defined by ***R*** = ***H*** ^*T*^, where:

$$H_{ij}=\left\{\begin{array}{cc} 1, & \;\;\;\;i=j,\;i=j+1\\ 0, & \text{otherwise} \end{array}\right.$$

this term encodes for temporal smoothness (Zhou et al., 2013).

## 3 RESULTS

**Figure 2:**
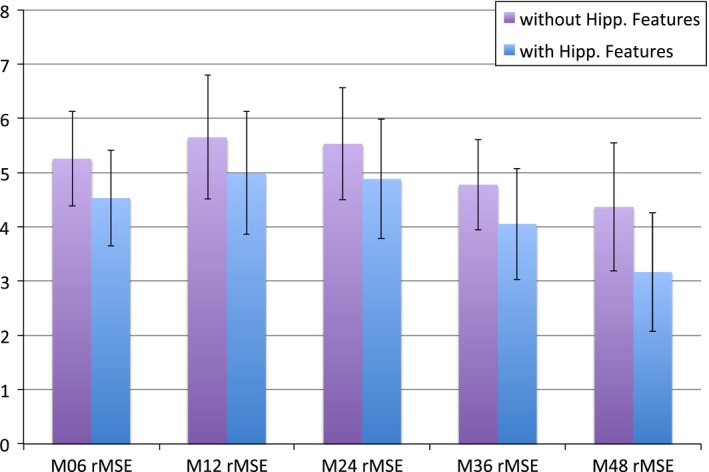
Bar Chart of the rMSE of predictions with and without hippocampal features by time points (6 months, 12 months, 24 months, 36 months, 48 months)

Predictions using hippocampus‐based feature maps outperform prediction without using feature maps as shown by quantitative measures such as nMSE, wR and rMSE. This was true across the board at all time points (see Table 2 and Figure 2). Our results show that incorporating large feature maps into sparsifying prediction tasks is not only possible but may improve results of the prediction.

**Table 2:** Comparison of model performance in predicting ADA Cognitive Score with and without mTBM features. The base set of features used were MRI information (305 features), Sex, Gender, Age, ApoE and baseline MMSE score. 7 Hippocampus feature maps were used: Mid Distance, 3 lambda values of the mTBM, magnitude of the Jacobian map and the first two eigenvalues of the Jacobian (See Table 1 and Figure 1 for more details)

	Without hippocampal features	With hippocampal features
nMSE	0.345 ± 0.075	0.249 ± 0.039
wR	0.828 ± 0.036	0.873 ± 0.022
M06 rMSE	5.259 ± 0.872	4.534 ± 0.883
M12 rMSE	5.653 ± 1.143	4.989 ± 1.134
M24 rMSE	5.532 ± 1.029	4.885 ± 1.094
M36 rMSE	4.777 ± 0.833	4.055 ± 1.024
M48 rMSE	4.367 ± 1.179	3.164 ± 1.091

The results shown are from 2 simulation experiments where data from ADNI was used to both train and test the cFSGL model. Experiment 1 uses demographic information (age and gender), FreeSurfer volumes and cortical thicknesses (326 features), the number of ApoE‐ ε4 alleles as well as a baseline MMSE score as features used in the model. Experiment 2 added the hippocampus features from each of the vertices of the hippocampus segmentation using FreeSurfer. The vertex information from the hippocampus was scaled down by a factor 10 using bi‐cubic interpolation to yield a total of 2100 features. 90 percent of the 624 subjects were used for training and the remaining 10 percent were used for testing. The results shown are from the 10 percent of our dataset allocated for testing. We calculated the root mean square error:

$$\text{rMSE}\left(y,\;\hat{y}\right)=\sqrt{\frac{||y-\hat{y}{\left|\right|}_{2}^{2}}{n}}$$

as well as a the correlation coefficient between the pairs of predicted values and actual values at each of the time points.

**Figure 3:**
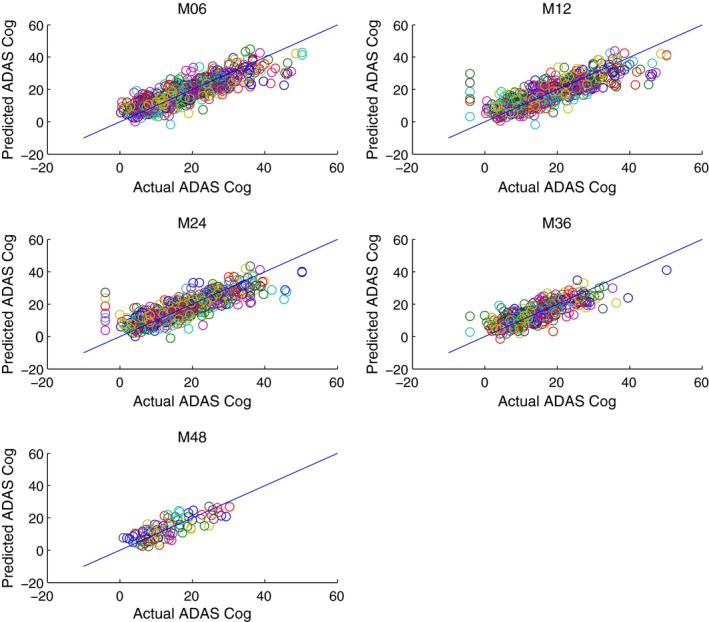
Prediction of ADAS Cog Score vs. Actual ADAS Cog Score *without using* mTBM features and only with MRI volumetric information, Age, Sex, Gender, ApoE and baseline MMSE score at M06 (6 months), M12 (12 months), M24 (24 months), M36 (36 months), M48 (48 months)

**Figure 4:**
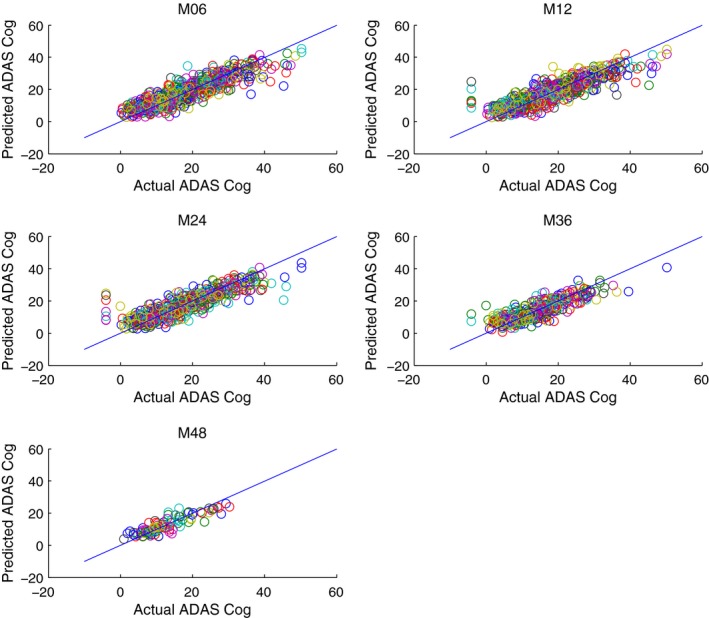
Prediction of ADAS Cog Score vs. Actual ADAS Cog Score *using* mTBM features together with MRI volumetric information, Age, Sex, Gender, ApoE and baseline MMSE score at M06 (6 months), M12 (12 months), M24 (24 months), M36 (36 months), M48 (48 months)

Table 2 shows how predictive performance has improved by incorporating hippocampus surface features into our dataset. There were improvements in predicting behavior outcomes at every time point. Moreover, by looking at the weights in predicting the behavioral outcomes, we may able to see which parts of the hippocampus feature maps are often used in predicting behavior. Figures 3 and 4 show that the raw prediction results from our multiple cross validation runs are reasonably distributed. These results were then used to calculate the different predictive performance measures such as Mean Square Error.

## 4 DISCUSSION AND CONCLUSIONS

By merging fused multi‐task learning that encodes temporal smoothing (Zhou et al., 2013) together with AD sensitive mTBM maps of the parametric hippocampus surface (Shi, Thompson, et al. 2013), we were able to get significant gains in future ADAS cognitive score prediction. These results are some of the highest performing predictions based on baseline data only and is consistent with our survey of other comparable studies (Zhou et al., 2013). There are two main findings in our work. First, we demonstrate surface mTBM when combined with other features, may significantly boost the statistical powers. This discovery is in line with many of our prior studies (Wang et al., 2011; Shi, Wang, et al., 2013; Wang et al., 2013; Shi et al., 2014). The newly combined surface statistics practically encodes a great deal of neighboring intrinsic geometry information that would otherwise be inaccessible, or overlooked. Second, cFSGL is an effective way to overcome the curse of the dimension with it's sparsity constraint. With proper tuning of parameters to match the features size, the sparsity constraint was also able to prevent overfitting, which tends to occur when using large number of features. Our work shed some light to future work to predict longitudinal neuropsychological changes and may help solve this challenging research problem.

One factor not addressed in this work is the effect of percentage of data used for training and testing. Previous work (Zhou et al., 2013) has shown that although there would be a decrease in performance measured with a smaller training set, the trends and relative performance remains comparable. We have also treated the parametric surface data, patient demographics and MRI volumetric information as one continuous information vector. It would be interesting to see if adding neighborhood information based on the location on the parametric surface would give us smoother and more realistic weights on the parametric surface and perhaps even better or more consistent results.

The current study also serves as an illustration of how machine learning methods can be used with whole parametric surfaces or even volumetric volumes such as in fMRI studies. However, as the number of voxels and vertex points increase, we again run into problems with the curse of dimensionality. To counter such problems, sparsifying penalties such as in cFSGL can be employed. However, without a reasonable starting weight, finding a reasonable solution that has the required sparsity can get computational intensive. One solution that we intend to explore is the use of stability selection in seeding the initial weights for the algorithm in a hierarchical approach to learning. We believe that this a reasonable way of leveraging prior information whilst allowing the algorithm to impose explore ensure temporal smoothness and sparsity.

As this is a model of an epidemiological system, we cannot ignore the investigator's selection of reasonable features. Moreover, the performance of the system is as interesting as the weights that yield the predictions.

### 4.1 Future Work

Our future work includes understanding the behavior of the weights across the parametric surface space as well as in time. Previous work has shown that stability selection may be a good fit for analyzing the feature weights on the model and may yield more information about the relationship between the deformation of hippocampal subfields and other clinical indicators during AD progression. Moreover, additional work can be done to investigate the specifics of how the addition of the large number of mTBM features has contributed to the final prediction results as well as the computational burden versus reward of the additional features.

## CONFLICT OF INTEREST

None declared.
